# Two decades of external peer review of cancer care in general hospitals; the Dutch experience

**DOI:** 10.1002/cam4.612

**Published:** 2015-12-29

**Authors:** Melvin J. Kilsdonk, Sabine Siesling, Rene Otter, Wim H. van Harten

**Affiliations:** ^1^ Department of ResearchNetherlands Comprehensive Cancer OrganisationUtrechtThe Netherlands; ^2^ School for Management and Governance/department of Health Technology and Services ResearchUniversity of TwenteEnschedeThe Netherlands; ^3^ The Netherlands Cancer InstituteAmsterdamThe Netherlands

**Keywords:** Accreditation, health care quality assessment, health care quality assurance, neoplasms, organization & administration, peer review

## Abstract

External peer review was introduced in general hospitals in the Netherlands in 1994 to assess and improve the multidisciplinary team approach in cancer care. This paper aims to explore the value, perceived impact, and (future) role of external peer review in cancer care. Semistructured interviews were held with clinicians, oncology nurses, and managers from fifteen general hospitals that participated in three rounds of peer review over a period of 16 years. Interviewees reflected on the goals and expectations, experiences, perceived impact, and future role of external peer review. Transcriptions of the interviews were coded to discover recurrent themes. Improving clinical care and organization were the main motives for participation. Positive impact was perceived on multiple aspects of care such as shared responsibilities, internal prioritization of cancer care, improved communication, and a clear structure and position of cancer care within general hospitals. Establishing a direct relationship between the external peer review and organizational or clinical impact proved to be difficult. Criticism was raised on the content of the program being too theoretical and organization‐focussed after three rounds. According to most stakeholders, external peer review can improve multidisciplinary team work in cancer care; however, the acceptance is threatened by a perceived disbalance between effort and visible clinical impact. Leaner and more clinically focused programs are needed to keep repeated peer reviews challenging and worthwhile.

## Introduction

Multidisciplinary team (MDT) work has become the standard in cancer care in the last decades as diagnostic and treatment options for various cancers grew 1. The importance of multidisciplinarity further increased due to a shift from disease‐focused management to a patient‐centered approach. The European Partnership for Action Against Cancer (EPAAC), launched by the European Commission in 2009, therefore identified multidisciplinary care as a key element in cancer care 2. Evidence on the impact of MDT‐work on clinical outcomes is sparse, partly due to difficulties in relating procedural‐ and organizational changes to the various possible benefits 3. A recent study in 13,722 breast cancer patients showed that improved multidisciplinary care was associated with improved survival and reduced variation in survival 4. While MDT‐work may seem self‐evident for specialized cancer centers or university hospitals, it is a more recent development and an organizational challenge for general hospitals 5.

In the Netherlands, regional Comprehensive Cancer Organisations were established in the eighties to disseminate specialized knowledge on cancer diagnosis and treatment and to improve service provision without having a treatment function themselves. They formed networks of health care professionals with the aim to improve cancer care and outcomes through research, guideline development, and implementation, knowledge exchange and organizational improvement, for instance, by promoting multidisciplinary care. The Comprehensive Cancer Organisation in the North of the Netherlands introduced an external peer review program in 1994 to review the multidisciplinary organization of cancer care in hospitals and provide relevant feedback for further improvement. The program focussed on the organization of cancer care within the general hospital setting. Over time, it evolved and also paid attention to patient centeredness and important (inter)national trends such as centralization. The primary focus remained on the organization of cancer care and the functioning of the multidisciplinary teams. Policies were reviewed but not checked for individual cases, such as compliance with policies for adjuvant chemotherapy or psychosocial care. Through self‐reviews, site visits and on‐site interviews the organization of cancer care in a hospital is evaluated and recommendations for improvement are given. Reviewers are specially trained clinicians and nurses from other hospitals. Hospitals participate voluntarily and are advised to participate in cycles of 4–5 years to ensure continuous cycles of quality improvement. Annex 1 gives more detailed information on the external peer review program. Similar programs have been introduced in other countries. In England, for example, National Cancer Peer Review (NCPR) was introduced as part of the National Cancer Programme in 2004, after a first round of peer review was conducted at a regional level in 2001 6. The English program focuses on performance for specific tumor groups, whereas the Dutch program primarily targets the multidisciplinary cancer care organization in hospitals as a whole.

Until now, we have published two peer‐reviewed studies on the effects of the Dutch peer review program for multidisciplinary cancer care 7, 8. Some positive effects were found on multidisciplinary colorectal cancer treatment but the outcomes needed to be interpreted with care due to possible confounding factors such as patient case‐mix and regional differences 7. No added value was found on multidisciplinary treatment of breast cancer, as regional factors seemed to exert a stronger effect on treatment patterns than hospital participation in external peer review 8. In general, (international) evidence on the effects of peer review on cancer care is sparse. In lung cancer, peer review was successful in stimulating quality improvement activities but improvements in treatment rates and patient experiences were small 9. Outside the field of cancer care, two studies report on the one‐ and three‐year evaluation of peer review for chronic obstructive pulmonary disease in the UK 10, 11. Findings after 3 years indicated an association with improved quality of care, service delivery, and changes that promote quality improvement 11. The one‐year evaluation revealed no differences showing that changes need a longer period to occur 10.

While evidence on external peer review is sparse, physicians worldwide are increasingly confronted with programs such as external peer review and accreditation. In this qualitative study, we aim to explore the role and impact of external peer review for multidisciplinary cancer care in the general hospital setting. In interviews with stakeholders, we evaluated the value, perceived impact and (future) role of external peer review in cancer care.

## Material and Methods

Semistructured interviews were conducted with stakeholders from general hospitals from two regions in the Netherlands that participated three times between 1994–2010 (North and Rotterdam region).The hospitals in these two regions have the longest experience in the program with three cycles of peer review in our study period. We excluded hospitals that merged in the study period, as this made it hard to reflect on recommendations and the impact of the program. From the two regions, all 26 qualifying hospitals were invited to participate in our study. Per hospital, we requested to interview a clinician involved in the treatment of cancer patients, an oncology nurse, and a representative from the board of directors or management. We aimed to interview at least two stakeholders per hospital. The following inclusion criteria were applied: (1) the interviewee was required to have participated in at least one peer review visit (preferably also involved in preparations for the program); (2) oncology nurses should have coordinating/organizational tasks; (3) the management representative had to be involved with cancer care management in the hospital. Participation in the interviews was voluntary and participants were not reimbursed.

The telephonic interviews were conducted in Dutch from May to October 2012 by the principal investigator (M. K). Participants were informed about the purpose of the study and how the data would be used. The interviews followed a fixed scheme. First, the motivation for participation was discussed, followed by the experiences with the program. This was discussed according to the chronological phases of the program: self‐review phase, the actual site visit and the aftermath. Consequently, the impact was discussed and examples of program effects were asked. To conclude, views on the role of the program in the future and possible improvements were asked. A list of general questions covering these topics was used. An overview of the main interview topics, questions, and the rationale behind the questions is presented in Table 1.

**Table 1:** Overview of interview themes with examples of questions and the rationale behind the themes and questions. In this table only the main ‘opening questions’ are presented

Interview topics	Main/opening question(s)	Rationale
Goals	What are your goals for participation in this program?	Explore if the incentive is organizational improvement, quality improvement or both
Experiences (program evaluation)	What are your experiences with the self‐assessment phase of the program and what was its value?What are your experiences with the actual site visit, what was its value and what was the added value after the self‐assessment?What is your opinion about the end‐rapport, did it reflect the state of cancer care in your hospital?	Experiences with the different program phases gives information on what the most important parts of external peer review programs are and when changes occur
Impact	In which areas did you experience a program impact?Can you give examples of program effects?	Answers give insights in how external peer review influences organization and care and which aspects of care are affected
Future	If the program would remain as it is now, would you participate again?How can the program be improved?	Does a program retain its value after three participations or does it need changes?

All interviews were recorded and transcribed verbatim, using word processing software by the principal investigator (M. K). All data were anonymized and interviewees were guaranteed that data would not be shared with the third party, allowing them to speak freely. The transcripts were analyzed by using ATLAS.ti software (version 7, www.atlasti.com). Using an inductive approach (organizing the data based upon common patterns or themes), the entire transcripts were coded. Answers were given a distinct code to get an overall impression of the results from the interviews. Relevant citations were selected as illustration per interview topic. The results and citations were discussed with other investigators (W. V. H, S. S). Despite the qualitative nature of this study, answers on specific questions, such as what the goals were to participate could be quantitatively analyzed. In these cases, frequencies of answers were used to determine their relative importance.

## Results

### Study population

Fifteen out of the twenty‐six invited hospitals participated in our study. Two hospitals reacted that they were either not able or not willing to invest their time in interviews, the rest did not reply to our invitation and gave no reason for not participating. In our study population of fifteen hospitals, it was not possible to interview two stakeholders in two hospitals; in one hospital, we could only interview a medical specialist and in another hospital, we only interviewed an oncology nurse. Additionally, in one hospital there was not a nurse available with sufficient experience with the program to participate. We could only interview four managers that met our criteria of being personally involved in at least one external peer review and involvement in cancer care (partly due to high management turnover). In total, data from 15 hospitals and 31 interviews were analyzed: 14 physicians (eight medical oncologists, four surgeons, one pulmonary physician, and one gynecologist), 13 oncology nurses, and four management representatives.

### Interview findings

#### Motivation

The motivation to participate could be coded into ten distinct codes or ‘buckets’ as can be seen in Table 2. We further categorized these codes into four main ‘themes’. The most frequently mentioned goal for participating in the program was to obtain feedback on the quality of organization and processes (coded 21 times). Clinical quality improvement is mentioned by a majority of interviewees (*N *=* *19) as a goal for participation, even though the program foremost has an organizational focus. Differences between physicians and nurses are seen in the positioning of cancer care which is an important goal especially for physicians (physicians: eight, nursing staff: two). By the position of cancer care, the interviewees mean that cancer care was given priority amongst a hospital wide range of services and became the joined responsibility for physicians, nursing staff, and management. This is illustrated by the next quote:

**Table 2:** Number of times a goal for participating in the peer review program was mentioned by different stakeholders. Interviewees could have had more than one goal

Goals/incentive for participation (themes)	Goals mentioned by interviewees	Physicians (*N *=* *14)	Nurses (*N *=* *13)	Management (*N *=* *4)	Total (*N *=* *31)
External motivation	Transparency	1	4	0	5
	Obligation	1	2	2	4
	See what external experts find important in cancer care	0	4	0	4
Organization of care	Quality test of organization and processes (see how well you are doing)	9	10	2	21
	Positioning of cancer care in the hospital (priority for management and/or physicians)	8	2	0	10
	Reveal organizational weaknesses (blind‐spots)	0	4	0	4
	Receive recommendations for improvement	2	2	2	6
	Re‐evaluate existing patterns in cooperation and communication	1	1	0	2
Clinical cancer care	Quality improvement of clinical cancer care	9	7	3	19
Future perspectives	Getting ready for changes in the future	1	0	1	2

As a medical oncologist you are not the only one in the web of physicians surrounding a patient. In every single case, a lot of physicians should communicate and cooperate. Sometimes one physician thinks this is more important than another. By participating in the program, you hope that attention is raised for everyone to see this necessity. [Oncologist]

#### Program experiences

The first phase of the external peer review, the self‐review phase, forced stakeholders to review the organization and cooperation within their own hospital. Nineteen respondents stated that self‐review through stating compliance to lists of organizational standards is a good method to discover weak points in their organization. It was said that changes already occurred in this preparation phase as existing policies were revised and corrected if necessary. Interestingly, all of these nineteen interviewees claim that the weaknesses were not totally unknown beforehand. The self‐review phase was also the most criticized part of the program. All interviewees answered that the investments (time and effort) were high. The questionnaires were criticized for being too theoretical, insufficiently suited for their individual situation and containing too many irrelevant and ‘obvious’ questions. It was mentioned three times that difficulties in answering questions sometimes resulted in giving ‘desired’ answers.

The actual site visit by peers, is valued highly, 20 respondents mentioned that this is the most important part of the program. Especially the dialog and opportunity to explain how they work was appreciated instead of only stating their compliance to a theoretical framework. Also, misinterpretations of answers given in the self‐assessment could be corrected. Almost all interviewees (*N *=* *29) stressed the importance of a committee consisting of peers because of the mutual understanding of problems that hospitals are faced with. Eighteen participants think that the composition of the review committee (three medical specialists and one oncology nurse) does not need to be altered. Suggested changes to the committee were to add an extra oncology nurse (*N *=* *2), a manager (*N *=* *2) or a professional from the psychosocial field (*N *=* *6). The rest of the interviewees had no opinion on this matter.

The final phase of the program starts with the end‐report based on the self‐assessment and findings of the site visit. The recommendations in the reports are generally regarded as a good reflection of the weaknesses and improvement points of the organization. All respondents answered that the recommendations are used in the cancer policy plans of their hospitals for the upcoming years. The reports are used to strengthen the position of the oncology services in negotiations with the board of directors and medical staff.

#### Perceived impact and examples of program effects

In order to gain more understanding of how the program impacted cancer care in hospitals, every stakeholder was asked to give examples of important effects of the program (if there were any). We coded the aspects of care that were influenced by the program. We found ten aspects of cancer care on which the program had a perceived impact. They are mentioned in Table 3 with the examples that were given by the interviewees. The frequencies of the answers give a sense of importance but because we asked for examples we did not use the frequencies to determine which aspect of care is most influenced by the program. Not mentioning an example does not mean that the program did not impact that aspect of care in their hospital.

**Table 3:** Examples of effects of the external peer review programs as mentioned by interviewees (*N* = 31) grouped per theme

Theme	Examples of impact given by the interviewees
Position of cancer care in hospital Oncology committee	Cancer care became a priority (*N* = 9) Large committees were formed with representatives from all disciplines treating cancer patients (*N* = 9) role of committee was officially established in hospital organization (*N* = 2) Committee got responsibility for policy making (*N* = 5) Functioning of committee improved (*N* = 3) Structural meetings were organized with board of directors and medical staff (*N* = 2)
Cooperation	Involvement of “smaller” disciplines such as gynecologists and urologists (*N* = 6) Improved communication between specialists and between specialists and nursing staff (*N* = 4) Improved communication with general practitioners (*N* = 1)
Multidisciplinary patient care meetings	Involvement smaller disciplines in the meetings (*N* = 3) Protocols on which patients have to be discussed (*N* = 14) Uniformity of reporting (*N* = 1)
Structure	Increased number of nursing staff (*N* = 6) Investments in ICT (*N* = 1)
Delivery of care	Advice on the introduction of integrated care pathways (*N* = 2) Concentration of chemotherapy administration within the hospital (*N* = 1)
Referral policies	Referral policies were made for rare tumors (*N* = 1) Official agreements were signed with other hospitals on which patients to treat and which to refer for further treatment. (*N* = 3)
Nursing staff	Introduction of specialized oncology nurses (*N* = 6) Education (*N* = 2)
Psychosocial care	Increased number of psychosocial staff (*N* = 6) Clarity on the role and positioning of psychosocial staff (*N* = 1) Introduction psychosocial protocols (*N* = 1)
Readiness for change	Organization is better prepared to adapt to future changes (*N* = 4)

A perceived impact on the position of cancer care within the hospital organization is expressed nine times. It was also mentioned ten times as goal for participation (Table 2). This seems to work two ways: as mentioned earlier, the participation itself creates attention and involvement. Secondly, the other examples of perceived impact also enforce prioritization of cancer care. For example, a perceived impact on the (role of the) oncology committee was expressed 21 times in total. Also, according to a total of nine interviewees the role of the committee within the hospital and policy making was formalized. The formation of multidisciplinary oncology committees with representatives from all disciplines that treat cancer patients was stimulated, especially in the first review rounds (most of the committees were small and not all disciplines were represented). This created an official structure within hospitals to advocate the interests of medical personnel and cancer patients in structural meetings with the board of directors and medical staff. Because the oncology committee was required to consist of representatives from all specialisms that treat cancer patients, ‘smaller specialisms’ like gynecologists and urologists became more involved which improved communication. There was also a more general perceived impact on the cooperation between physicians and between physicians and nursing staff (*N* = 4). Concerning clinical care, most impact was experienced on the multidisciplinary patient care meetings. Fourteen interviewees mentioned an example of impact on these meetings. Due to the recommendations, multidisciplinary patient care meetings were professionalized, protocols were developed on which patients should be discussed in these meetings and reporting was standardized. The program required weekly meetings where every newly diagnosed cancer patient was discussed in the multidisciplinary team to improve shared decision‐making. Less frequently mentioned examples of program impact concern impact on structure, delivery of care, psychosocial care, nursing staff, referral policies, and future perspectives. These include increased numbers of staff, better integration of psychosocial care and advice on the introduction of integrated care pathways (Table 3).

Interviewees found it difficult to single out the effects of the program. Investments in extra oncology nurses can be contributed to clear recommendations in the final reports of the program, but other (clinical) effects of the program do not stand on their own. This is illustrated in the next citation:

Every patient has an individual case‐manager now. That would probably have been established anyway, but because of the recommendation of the program it might have been introduced earlier. Yes, I think that had an impact. Another example is the multidisciplinary patient care meeting. They needed to be held more often and larger groups of patients needed to be discussed. I think that this would have been realized anyway because of the national guidelines and not only because of the program, but it certainly influenced it. [Oncology nurse]

#### Future role and improvements of the program

All hospitals in our study population participated in three review rounds. The program started with a strong focus on basic organizational requirements and evolved from there with more emphasis on professional quality and care pathway organization. The organizational focus remained to be a source of frustration. Twenty interviewees expressed their concerns on having to repeat all the organizational items in the self‐review and site visit in a fourth participation round. As a result, only 12 persons would still find a fourth participation worthwhile without major changes in the program. This suggests that a mismatch between the investments and experienced benefits is a potential pitfall for the program.

Suggestions for improvement that were given are as follows:

1. move beyond the basic organizational conditions and focus on the actual delivery of care
2. decrease the time investments needed for self‐assessment.
3. more emphasis on current and future requirements in oncology.
4. focus on one or two specific types of cancer.
5. give hospitals the opportunity to indicate on which parts of the care process they would like to receive in‐depth feedback.
6. strengthen the patients’ perspective compared to the organizational perspective.

Opinions varied whether the focus of the program should remain on the entire cancer care organization or on specific types of cancer. Advantages mentioned of a tumor‐specific program were a better focus on actual care, less time‐consuming preparations, and the possibility to assign clinical experts as reviewers.

Disadvantages of a tumor‐specific program were also mentioned. There are already multiple organizations that have developed registrations and clinical audits for specific diseases. Because of this, there is a risk of an overkill of external assessment programs. Other interviewees mentioned that the necessity to look at the entire organization does not change; weak points in the organization mostly concern aspects of cooperation and communication, which can be easier tackled through an organization focussed program.

## Discussion

It can be carefully concluded that the external peer review program for multidisciplinary care in the Netherlands had a perceived positive impact on several aspects of cancer care. Most frequently mentioned were the internal positioning of cancer care, formation and role of oncology committee and multidisciplinary team meetings. Part of the experienced impact could be attributed directly to the program based on recommendations in the final reports. Interviewees were hesitant to attribute more clinically oriented effects to the program alone, as many factors can be of influence. Although the program has an organizational focus, improvement of clinical care is mentioned as a motivation to participate almost as often as organizational improvement. Criticism was also raised, particularly on the repeated organizational focus and missing links with clinical care (most outspokenly in the self‐review phase). Nineteen interviewees mentioned that while the self‐review uncovers organizational weak spots, they were not entirely unknown beforehand. It therefore seems that the value lies in directing attention to these weak spots. The actual site visits were regarded as the most important part of the program because of the dialog that occurs with their peers from the review committee.

It is difficult to prove a direct impact of external quality improvement programs. A previous mixed‐method study on accreditation also struggled to answer the question. Performance on accreditation was found to be an accurate reflection of contextual organizational factors believed to be important in enabling or inhibiting quality of care and continuous quality improvement 12. A French study on the impact of accreditation used a hypothetical model in which accreditation is seen as an agent of change 13. These studies complement our findings that external peer review is one of multiple factors that initiates change and contributes to a better organization that can lead to quality improvement.

The external peer review program for multidisciplinary cancer care was introduced to strengthen and support the (introduction of) multidisciplinary team work. There are two main categories of barriers to effective cancer care coordination. Firstly, those barriers that are a result of an ineffective team (recognition of health professional roles and responsibilities, transition of care, inadequate communication), and secondly barriers that are the result of inadequate resources, including managing scarce resources and inequitable access to health services 5. Our results especially show a perceived program impact on those barriers resulting from ineffective team work. Examples of the perceived impact revealed a transferral of responsibilities from individual physicians to multidisciplinary teams consisting of physicians, nursing and supporting personnel. This lead to prioritization of cancer care, improved communication, and a central position of cancer care within general hospitals.

Contrary to our finding that the site visit is the most valued part of the program, previous research identified the self‐review as the most important 13. This might be due to the fact that the review committee consists of peers, which creates mutual understanding. It also implies that stakeholders value an approach that is not overly focussed on standards. Touati and Pomey described this earlier and drew parallels between accreditation and a management model of “commitment based management” instead of “control based management” 14. Leaving a philosophy of control and adapting a philosophy of commitment provides greater room for autonomy and creativity of the reviewed hospital and stakeholders. This approach to quality management requires a challenging balance of improvement dynamics with standardization and assurance, but can be used to prevent the perception of external quality assessment programs as coercive tools of over‐standardization 15. The disbalance between effort and effect threatens the legitimacy of the program. Possibilities for improvement of the program are mainly to move away from basic organizational requirements after a first or second round and focus more on actual clinical care. This can be achieved by making better use of the information from previous peer reviews. When it has been established that the basic organizational requirements were met, new peer reviews can pay attention to other aspects of care. This might create leaner and more flexible programs.

Our study has several limitations. There will always be a certain degree of interpretation when working with qualitative data. We tried to minimize this by coding the transcripts with specialized software. The frequencies are mentioned to give a sense of importance. We were cautious to use this to make statements on the impact of the program. The frequency counts in Table 2 and 3 are unbalanced which makes it difficult to draw firm conclusions. It does give a general overview of motivation and perceived impact. Although we interviewed representatives from 15 different hospitals, a larger study population might have revealed more details (although we experienced a considerable degree of saturation). We could only interview four management representatives due to a high management turnover and because managers were not always involved in the process before the actual site visit. Eleven hospitals that were invited did not participate which might have resulted in a selection bias. The hospitals were invited because their regions were the first to implement the program. Therefore, our findings only represent hospitals with multiple participations in the program. The interviews were not done after each peer review but retrospective after three cycles of review which might cause a fading of memories. Hospitals that participate for the first time might find it more useful to focus on more basic organizational conditions. However, our research is unique in investigating organizations with multiple participations and our results show a decreasing acceptance for external peer review if a program keeps focussing on organizational standards.

## Conclusion

Organizational external peer review can be an appropriate method for general hospitals to improve multidisciplinary team work in cancer care. In general hospitals, it can help in the internal positioning of cancer care and to improve structures and processes that encourage multidisciplinary team work. Our findings suggest that, concerning actual clinical care, external peer review has an indirect impact by influencing the multidisciplinary patients meetings, numbers of staff etc. The acceptance of a program that primarily focuses on organizational requirements decreases after multiple participations. As a majority of interviewees participate to improve both organizational and clinical care, we argue that moving from an organizational focus to clinical cancer services in future participations can keep external peer review worthwhile and challenging.

## Conflict of Interest

The authors declare no conflicts of interests.
